# Pheochromocytoma presenting with severe abdominal pain and abnormal liver enzymes

**DOI:** 10.1002/ccr3.4640

**Published:** 2021-08-15

**Authors:** Mhd Baraa Habib, Mohamed Abdelrazek, Sali Alatasi, Mouhand F. H. Mohamed, Hamda Ali, Mohamad Khair Hamad

**Affiliations:** ^1^ Internal Medicine Department Hamad Medical Corporation Doha Qatar; ^2^ Radiology Department Hamad Medical Corporation Doha Qatar; ^3^ Pathology Department Hamad Medical Corporation Doha Qatar; ^4^ Endocrinology Department Hamad Medical Corporation Doha Qatar

**Keywords:** high transaminase, pheochromocytoma, severe abdominal pain, unusual presentation

## Abstract

Pheochromocytoma can present with right hypochondrial pain, elevated liver enzymes, and a misleading appearance on ultrasound scan mimicking hepatic mass due to the proximity of adrenal masses to the liver.

## Graphical abstract

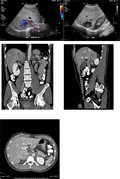

## 1 INTRODUCTION

Abdominal pain is a rare manifestation of pheochromocytoma. We report a young gentleman who presented with severe abdominal pain and elevated liver enzymes. The ultrasound scan (US) initially misled us toward hepatic mass, but computed tomography (CT) showed an adrenal mass. Furthermore, urine normetanephrine level was high, which established the diagnosis of pheochromocytoma.

Acute abdominal pain is a common symptom in patients presenting to the hospital worldwide. The differential diagnosis of abdominal pain is quite wide. The history, physical examination, laboratory workup, and imaging usually give clues and lead to the underlying cause. However, sometimes the presentation can be challenging, and the diagnosis may be delayed.^1^

Pheochromocytoma is a rare catecholamine‐secreting neoplasm. It is probably occurring in less than 0.2 percent of patients with hypertension.^2^ It mostly manifests in the fourth to the fifth decade with equal distribution between males and females. Pheochromocytoma classically manifests as episodic headache, sweating, and tachycardia. An increasing proportion of patients are asymptomatic, and the adrenal mass is incidentally discovered while being evaluated by imaging modalities for other reasons. Hypertension is a very frequent sign of pheochromocytoma.^3^, ^4^, ^5^ Gastrointestinal manifestations such as nausea, vomiting, and constipation are less common in the context of pheochromocytoma. Abdominal pain is a rare manifestation with only a few reported patients presented with severe abdominal pain.^6^ Basic laboratory tests may show increased erythrocyte sedimentation rate, hyperglycemia, and leukocytosis. Elevated liver enzymes were mentioned in one report.^2^ Biochemically, the diagnosis is established by elevated levels of metanephrine and/or normetanephrine level in serum or urine, followed by radiological imaging for tumor localization. The definitive treatment is the surgical removal of the neoplasm.^2^

In this case, we describe a patient who presented with severe abdominal pain associated with elevated liver enzymes and ultrasound suggestive of hepatic mass, which deviates our thinking initially toward a liver pathology. Later, a CT scan revealed a right adrenal mass adjacent to the liver; subsequently, the biochemical test confirmed pheochromocytoma.

## 2 CASE REPORT

A 41‐year‐old patient known to have hypertension presented with a 3‐day history of right upper quadrant abdominal pain. Initially, the pain was mild but progressed rapidly and became very severe. It was slightly improved with paracetamol. It was associated with nausea and one episode of vomiting. The patient denied any fever, heartburn, or diarrhea. He was on regular amlodipine and perindopril. On physical examination, blood pressure was 190/120 mmHg with a heart rate of 89 per minute. The abdomen was soft and lax with mild tenderness in the right upper quadrant, but no masses, organomegaly, or flank tenderness. He was requiring fentanyl to control the pain. Laboratory tests showed normal renal function and serum electrolytes but high transaminases (Alanine aminotransferase [ALT] = 161 U/L, Aspartate aminotransferase [AST] = 80 U/L; Table 1). Serum amylase and lipase were within normal. For the evaluation of the high transaminases, a viral hepatitis panel was done which came back negative.

**TABLE 1:** Laboratory tests results upon admission

Detail	Value w/units	Normal range
Adjusted calcium	2.45 mmol/L	2.20–2.60
Urine 24 metanephrines	0.4 μmol/24 h	0.0–1.9
Urine 24 nor‐adrenaline	13,005 nmol/24 h	0–570
Urine 24 nor‐metanephrines	35.0 μmol/24 h	0.0–4.5
Urine 24 vanillylmandelic acid	102.8 μmol/24 h	0.0–33.0
Cortisol	539.0 nmol/L	
Dehydroepiandrosterone sulfate	4.410 μmol/L	2.410–11.600
Aldosterone	355.0 pmol/L	48.9–644.4
alpha‐fetoprotein	5 IU/ml	0–6
Entamoeba histolytica antibody	Negative	
Echinococcus antibody	Negative	
Hepatitis B Core Ab IgM	Non‐reactive	
Hepatitis A Ab IgM	Non‐reactive	
Hepatitis B surface antigen	Non‐reactive	
Hepatitis C Ab	Non‐reactive	
Total protein	74 gm/L	60–80
White blood cell count	10.5 × 10^3^/μl	4.0–10.0
Hemoglobin	15.6 gm/dl	13.0–17.0
Urea	2.8 mmol/L	2.5–7.8
Creatinine	74 μmol/L	62–106
Sodium	139 mmol/L	133–146
Potassium	3.5 mmol/L	3.5–5.3
Bicarbonate	26 mmol/L	22–29
Alanine transaminase	161 U/L	0–41
Aspartate aminotransferase	80 U/L	0–40
Amylase‐P	32 U/L	13–53
Lipase	38 U/L	13–60
C‐reactive protein	8.0 mg/L	0.0–5.0
Lactic acid	1.9 mmol/L	0.5–2.2
pH Venous	7.40 mmHg	7.35–7.45

**FIGURE 1:**
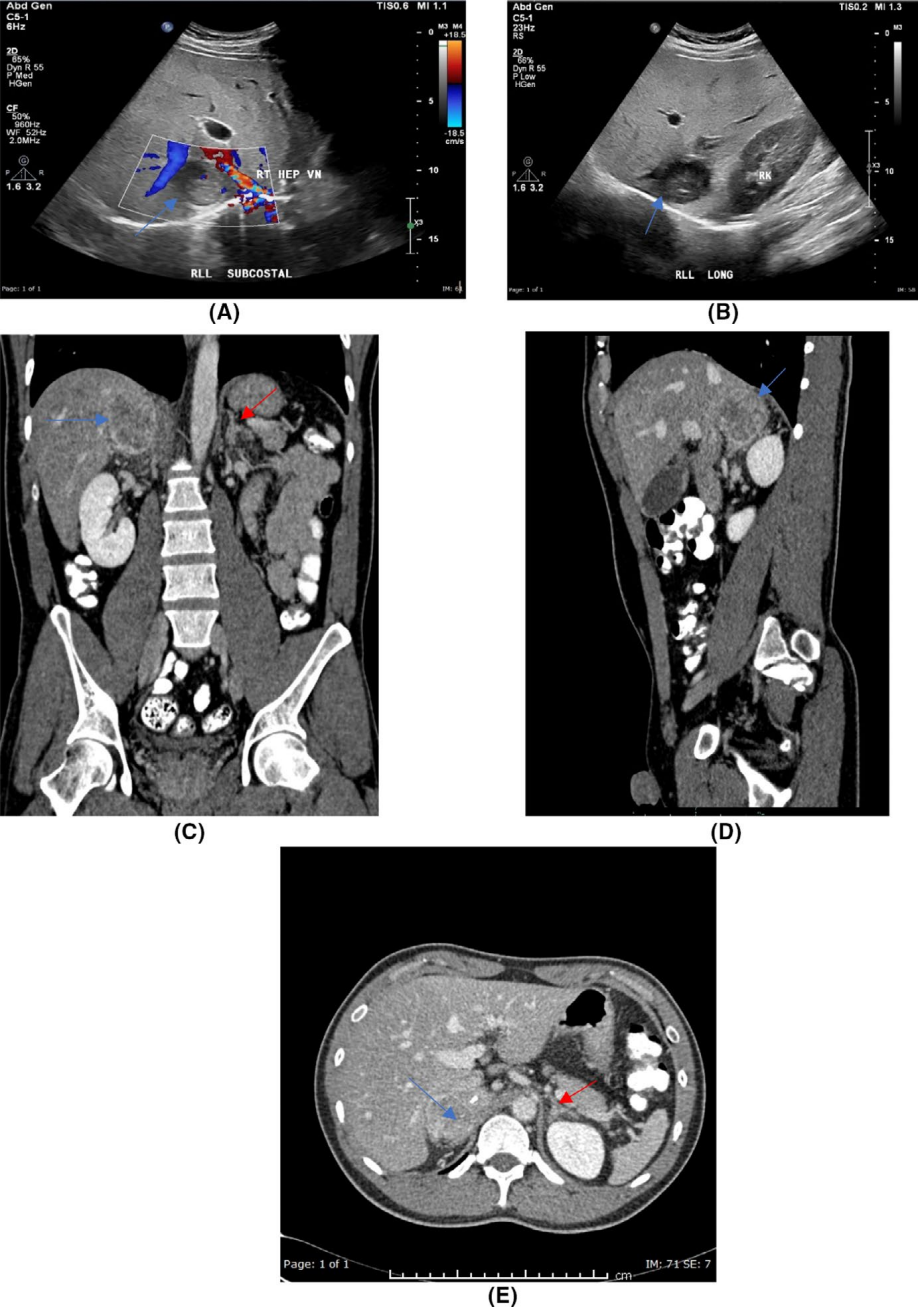
Radiology images (A, B) Abdominal ultrasonography showing focal hypoechoic lesion with mild vascularity by color Doppler related to superior pole of the right kidney and inseparable from right lobe of the liver (Blue Arrows) (C–E) Coronal, sagittal, and axial post contrast computed tomography image of the abdomen showing heterogeneous mass measuring 38 × 34 × 46 mm in the right supra renal gland with central areas of necrosis and calcification inside (blue arrows). The left supra renal gland in normal (Red Arrow in C, E)

**FIGURE 2:**
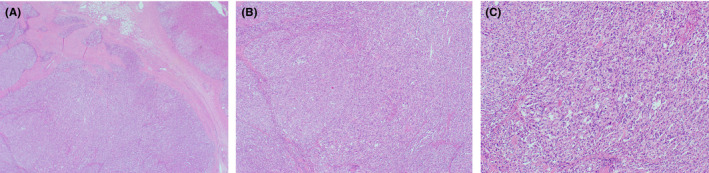
Pathology sections (A) A tumor in the medulla of the adrenal gland that is arranged in nests (Zellballen) and surrounded by compressed adrenal gland. (B) Higher power view of the nested patter of the tumor. (C) The tumor cells have abundant gray and granular cytoplasm, and nuclei with granular chromatin (salt and pepper chromatin)

Ultrasound abdomen showed a focal hypoechoic lesion in the right lobe of the liver (Figure 1). CT of the abdomen revealed heterogeneously enhancing right adrenal gland mass, with central hypodensities. It is seen indenting segment VI of the liver and causing mild anterior displacement of the inferior vena cava. Liver abnormality was detected (Figure 1). 1 mg dexamethasone suppression test, plasma aldosterone, plasma renin activity, and 24‐h urine metanephrine and normetanephrine were measured for functional evaluation of the adrenal mass. The laboratory tests were only remarkable for high 24‐h normetanephrine 35.0 μmol/24 h (normal range 0.0–4.5), which suggests pheochromocytoma as the cause of the adrenal mass. For blood pressure control and preoperative preparation, the patient was started on prazosin, the dose was titrated up gradually, and then, propranolol was added. He underwent right adrenalectomy. The tissue pathology showed pheochromocytoma (Figure 2). The patient had a follow‐up visit 2 months after the surgery, he was asymptomatic, and his liver enzyme levels returned to normal (ALT = 35 U/L, AST =34 U/L).

## 3 DISCUSSION

Abdominal pain is a very common manifestation in patients presenting to the emergency department. A lot of medical and surgical diseases can present with abdominal pain. Biliary and hepatic etiologies cause right upper quadrant pain syndromes. Common causes include gallstones, acute cholecystitis, acute cholangitis, hepatitis, liver mass or abscess, Budd‐Chiari syndrome, and sometimes basal pneumonia.^7^ Abnormal liver enzymes in combination with severe abdominal pain raise the suspicion of hepatic pathology, although other illnesses such as diabetic ketoacidosis, and peritonitis also were reported.^8^

Although pheochromocytoma usually presents with episodic headache, sweating, and tachycardia, a few cases reported patients presented with acute abdominal pain.^3^, ^9^, ^10^, ^11^ The mechanism of pain was mainly due to mass effect on the liver, hemorrhagic necrosis, or rupture of the tumor.^10^, ^11^

High transaminases in association with pheochromocytoma were described in previous cases.^6^, ^12^ A study suggested that the overproduction of catecholamines increases the resistance of liver arterioles and veins and decreases the blood flow and oxygen supply to the liver, which may result in abnormal liver function and high‐liver enzymes.^13^

In our patient, the clinical picture was not supporting the usual common causes of abdominal pain.

Pheochromocytoma, as the cause of both abdominal pain and the elevated liver enzymes, is supported by the resolution of the pain and normalization of the liver enzymes after adrenalectomy. The mass effect and overproduction of catecholamines from the pheochromocytoma could explain this unusual presentation.

## 4 CONCLUSION

Pheochromocytoma is a rare tumor, which can present with unusual manifestations such as severe abdominal pain with elevated liver enzymes. A high level of suspicion should be kept in mind to avoid any delay in diagnosing such a serious but treatable disease.

## CONFLICT OF INTEREST

The authors report no conflict of interest.

## AUTHOR CONTRIBUTIONS

MBH: literature review and manuscript writing. MA: radiology imaging. MFM: literature review. HA & MKH: mentorship, literature review and manuscript revision.

## INFORMED CONSENT

Due to the COVID‐19 situation and its impact on direct patient contact, only verbal consent was obtained to publish this case.

## Data Availability

The data that support the findings of this study are available from authors, MBH and MKH, upon reasonable request.
